# Prospective multicentre study of patients with cutaneous metastases from breast cancer treated with electrochemotherapy

**DOI:** 10.1007/s10585-025-10350-5

**Published:** 2025-05-29

**Authors:** Francesco Russano, Giacomo Corrado, Antonio Bonadies, Emilia Migliano, Raimondo di Giacomo, Emanuela Esposito, Claudio Zamagni, Ada Ala, Luca Campana, Tommaso Fabrizio, Matteo Ghilli, Dante Palli, Mariuccia Renne, Roberta Cabula, Fabio Pelle, Barbara Silvestri, Maria Vittoria Dieci, Valentina Guarneri, Marco Rastrelli

**Affiliations:** 1 Soft-Tissue, Peritoneum and Melanoma Surgical Oncology Unit, Veneto Institute of Oncology, IOV-IRCCS, Padua, Italy; 2 Dipartimento Scienze della Salute della Donna, del Bambino e di Sanità Pubblica, UOC Ginecologia Oncologica, Fondazione Policlinico Universitario A. Gemelli, IRCCS, Rome, Italy; 3 San Gallicano Dermatological Institute IRCCS, Rome, Italy; 4 Chirurgia Oncologica di Senologia Istituto Nazionale Tumori IRCCS Fondazione G. Pascale, Naples, Italy; 5 IRCCS Azienda Ospedaliero-universitaria di Bologna, Bologna, Italy; 6 Breast Unit, Città della Salute di Torino, Torino, Italy; 7 Department of Surgery, Manchester University NHS Foundation Trust, Manchester, UK; 8 IRCCS-Centro di Riferimento Oncologico della Basilicata, Rionero in Vulture, Italia; 9 Breast Centre, University Hospital of Pisa, Pisa, Italy; 10 Breast Unit, UOC Chirurgia Generale, Piacenza, 29121 Italy; 11 Chirurgia Senologica/UOC Chirurgia Generale AOU “R. Dulbecco” Catanzaro, Catanzaro, Italy; 12 Ospedale Oncologico “A.Businco”- ARNAS Cagliari, Cagliari, Italy; 13 Chirurgia Senologia Istituto Tumori Regina Elena, Roma, Italy; 14 Oncology and Haematology Unit, Azienda Unità Sanitaria Locale Socio-Sanitaria (AULSS) 3 Serenissima - Ospedale di Mirano, Venice, Italy; 15 Department of Surgery, Oncology, and Gastroenterology, University of Padua, Padua, Italy; 16 Oncology 2, Veneto Institute of Oncology IOV-IRCCS, Padua, Italy

**Keywords:** ECT, Breast cancer, Cutaneous metastases, Subcutaneous metastases, Electroporation

## Abstract

Electrochemotherapy (ECT) is a local treatment combining chemotherapy with electroporation. This prospective multicentre study aimed to evaluate the efficacy of ECT in the treatment of patients with skin metastases from breast cancer and confirm whether “luminal A-like” tumors are more responsive to treatment. One-hundred and ninety-five patients were included in the analysis. 55% achieved complete response, 27% partial response (objective response OR 82%); 12% stable disease and 5% experienced progressive disease. The analysis by tumor phenotype showed a significant better response rate in Luminal A-like (*p* = 0.0060) and Luminal B-like (*p* = 0.0271) groups compared to Triple-Negative. Patients were divided into 4 groups based on the number and size of cutaneous metastases. Higher response rate was observed in patients with small (≤ 3 cm), single or multiple, metastases (OR rate 95% and 90%, respectively); larger tumors (> 3 cm) showed an OR rate of 85%. Tumor response was not affected by the presence of distant metastases, whereas patients with large cutaneous lesions and distant metastases showed a OR rate of 58%. One-year local progression-free survival (LPFS) was 86% (C.I. 82-89%). In the multivariate analysis, patient age and response to ECT were significantly associated with longer LPFS. This study confirms the efficacy of ECT in small-volume cutaneous metastases from breast cancer regardless the presence of systemic disease and suggests higher efficacy in patients with luminal A- and luminal B-like tumors. ECT can be utilized not only as a palliative measure but also as an alternative treatment for patients not eligible for standard treatments, or in combination with them. Trial registered on https://clinicaltrials.gov/study/NCT06683404 (date of registration 11/11/2024) retrospectively registered.

## Introduction

Breast cancer is the most common cancer affecting women worldwide, resulting in significant morbidity and mortality rates [1]. While advances in treatment modalities such as surgery, chemotherapy, radiotherapy and targeted therapy have improved patient outcomes, there is still a need for innovative approaches to enhance treatment efficacy, especially for locally advanced or recurrent disease [2–4]. The current treatment algorithm for advanced breast cancer relies on selected, systemic treatments tailored to the biological subtype of the disease, which may be complemented by locoregional interventions based on the affected sites and clinical needs [5].

In patients with metastatic breast cancer, the skin is affected in 5 to 30% of cases [6]. Although relatively infrequent in absolute terms, the onset of skin metastases generally represents an unfavourable prognostic factor due to the synchronous progression of diseases in other locations, as well as a challenging therapeutic management condition [7]. Even in the most favourable scenarios - where the disease remains confined to a locoregional level - skin metastases often result in a decline in patients’ quality of life due to their psychological impact and associated symptoms such as pain, ulceration, bleeding, infection. Therefore, it is essential that patients are offered a timely and effective treatment. Locoregional treatments such as surgery or radiotherapy may be applied to obtain local disease control. Systemic treatment options include chemotherapy, endocrine therapy, targeted agents and immunotherapy, tailored according to the breast cancer subtype. However, the aforementioned approaches may prove demanding or ineffective due to the extent of the disease, drug resistance, overly invasive procedures for the advanced stage of metastasis, or involvement of highly sensitive areas of the body. In this context, electrochemotherapy (ECT) may emerge as a valid and effective alternative [2, 6].

ECT is a relatively novel therapeutic modality that combines chemotherapy drugs with electroporation, a technique that transiently increases cell membrane permeability through the application of short, high-voltage electrical pulses. This approach facilitates enhanced uptake of chemotherapy agents (e.g. bleomycin and/or cisplatin) by cancer cells, thereby improving their cytotoxic effects while minimizing systemic toxicity [8]. The ECT treatment has been applied to several conditions: cutaneous and subcutaneous metastases of any histological origin, but also in visceral settings, such are liver [9], bone [10], vulva [11], pancreas [12], and recently in the treatment of vascular anomalies [13, 14]. Since early clinical experiences, ECT has shown promising results in patients with breast cancer [15]. In 2012, a phase I/II study [16] investigated the safety and efficacy of ECT in patients with chest wall recurrence from breast cancer. The study reported an effective chest wall control in about one third of patients with minimal systemic toxicity, suggesting that ECT could be a valuable treatment option for patients with unresectable or recurrent breast cancer. In 2015 a multicentre retrospective study published by the Italian Senology Group for Electrochemotherapy (Gruppo Italiano Senologi per l’Elettrochemioterapia GISEL) analysed 125 patients with skin metastases from breast cancer [17]. Data from this study showed a response rate to ECT of 90%, with 58% of patients reporting a complete response (CR). Based on these data and stratifying patients by intrinsic subtypes indicated by the St. Gallen classification [18, 19], patients with “luminal A-like” tumours exhibited a higher CR rate compared to the other subtypes (73.9% vs. 54.7%, *P* = 0.02).

This prospective multicentre study was designed to evaluate the treatment of skin metastases from breast cancer using ECT and identify predictive factors of response. In particular, the study aimed to confirm whether patients with “luminal A-like” tumors are more responsive to ECT.

## Materials and methods

### Patients’ recruitment criteria

Patients were collected from 12 centres belonging to the GISEL study group, based on the following inclusion criteria: patients with skin metastases from breast cancer that cannot be surgically removed, no indications for treatment with radiotherapy, patients who are not candidates or not completely responsive to systemic oncological treatments, maximum depth of the lesion < 3 cm, life expectancy > 4 months, normal hepatic and haematologic function, ECOG performance status within 0–2. Patients were excluded if they met any of the following criteria: known allergic reactions to bleomycin or cisplatin, maximum cumulative bleomycin dose already overcame (250000 IU/m^2^), severe hepatic or renal failure, epilepsy, patients with implanted pacemakers (for chest wall treatments) severe cardiac arrhythmias, pregnancy and breastfeeding, unavailability for follow-up visits, impaired respiratory function (acute or chronic). Moreover, patients receiving concomitant systemic treatment or who initiated a new systemic antineoplastic treatment after electrochemotherapy were included in the analysis. Patients have been selected for inclusion by an institutional multidisciplinary team (MDT) in each hospital.

The study was registered on 11/11/2024 (https://clinicaltrials.gov/study/NCT06683404). The study was first approved by the ethics committee of the Veneto Institute of Oncology (CESC-IOV) (protocol code: 4352, date of approval: 10/03/2017). The respective institutional review boards and ethic committees of the participating institutions then approved the study. The study was conducted in accordance with the 1964 Helsinki Declaration and its later amendments.

Histologic diagnosis, immunohistochemical analysis, and fluorescence in situ hybridization for HER2 gene amplification (in case of HER2 2 + score by immunohistochemistry) were performed according to international guidelines. Surrogate subtypes were defined according to the criteria established by the St. Gallen International Breast Cancer Conference [19].

### ECT treatment

Electrochemotherapy sessions were performed according to The European Standard Operative Procedures of Electrochemotherapy (ESOPE) for all patients [20, 21]. Accordingly, the dose and route of drug administration, usually bleomycin, were adopted based on the number and size of tumours: either intratumorally injection, or intravenous infusion was used. The procedure was scheduled in a day-hospital regimen, and patients were usually discharged after an observation period of 24 h.

Patients were followed according to the protocol up to 12 months, and then for varying periods depending on the centre and availability of the patients themselves.

### Toxicity

Several loco-regional symptoms were recorded both prior to ECT treatment and during follow-up visits and these included: pruritus, purpura, rash, skin atrophy, skin induration, hyperpigmentation, avascular necrosis, skin ulceration, body odour, superficial soft tissue fibrosis, bleeding. Symptom severity was graded according to CTCAE version 5.0 and were grouped into: not occurring, mild (grade I/II) or severe (grade III/IV). In addition, pain intensity was reported by the patient using the Visual Numeric Scale (VNS) ranging from 0 (no pain) to 10 (maximum pain). All data on VNS were recorded before procedure, immediately after and at each follow-up visit.

### Response evaluation

The evaluation of local tumour response was assessed by measuring the size of the treated lesions. The response for each target lesion was recorded at every follow-up visit and data from the first assessment (1 month after ECT), confirmed at 2 to 3 months were considered for local tumor response, in accordance with the Modified Response Evaluation Criteria in Solid Tumours (RECIST) [22]: complete response (CR) was defined as disappearance of the target lesion; partial response (PR) with at least 30% decrease in the diameter of the target lesion; progressive disease (PD) with at least 20% increase in the diameter of the target lesion; and stable disease (SD) with neither sufficient shrinkage to qualify for PR nor sufficient increase to qualify for PD. In some cases, with ulcerated tumours, evaluation was not possible because of crust formation. Data on local progression-free survival (LPFS) and overall survival (OS) were also collected.

### Statistical analysis

The primary objective of the study was to confirm the results obtained in the previous multicenter retrospective study from the GISEL group. In that study, breast cancer patients with “luminal A-like” disease who underwent ECT with bleomycin achieved a significantly higher complete response rate compared with other surrogate subtypes, defined according to the St. Gallen classification (73.9% vs 54.7%, P = 0.02). In the present study we intended to verify, on a large perspective series, if the rate of complete response in patients with “luminal A-like” breast cancer is significantly higher than in the other subgroups. The percentage of women with “luminal A-like” breast cancer in the previous study was 18.4%. We hypothesized to have the same patient distribution in the prospective study. To test the hypothesis of a significant difference between “luminal A-like” tumors and the other surrogate subtypes with a statistical power of 90% and a significance level of 5%, 351 patients were required (64 in the “A luminal-like” group and 287 in the group non-“luminal A-like”). Considering a dropout of 15%, the target accrual was 404 patients.

Descriptive methods were employed for statistical analysis: continuous variables were described using the mean, standard deviation, median value and range, while categorical variables were reported by absolute number and percentages. Comparisons among groups were conducted using the ANOVA test for continuous variables and Chi square-test for trend analysis with categorical variables.

Univariate analysis was performed for each subgroup using a logistic regression model to assess the objective response rate of the investigated variables. Significant variables were included in the multivariate model to evaluate their independent influence.

Local tumour control was expressed as local progression-free survival, which was the time from ECT up to the date of relapse or progression or last follow-up. Survival curves for local progression-free survival (LPFS) and overall survival (OS) were calculated using the Kaplan–Meier model. Univariate and multivariate Cox regression analysis was performed to identify variables affecting LPFS and OS. Significance of tests was reported with p-value, where a value < 0.05 was considered as statistically significant. All the analysis has been conducted by means of NCSS^®^ version 9.16 software.

## Results

Between May 2017 and December 2022, 204 patients were recruited and included in the database from 12 Italian Senologic Centres (Padova, Roma, Napoli, Bologna, Torino, Rionero in Vulture, Pisa, Piacenza, Catanzaro, Cagliari, Mirano). The study was started in May 2017 with a target sample size of 404 patients and was closed in December 2022 because of slow accrual, mainly due to COVID pandemic. Nine patients dropped out of the study within the first month after recruitment due to the following reasons: death due to disease progression (*n* = 6), systemic progression of disease (*n* = 2), lost to follow-up (*n* = 1). In conclusion, 195 patients were treated and evaluated for their response to ECT. The mean patients age was 66.8 ± 13.3 years (median 69 range 31–92) and mean time between breast cancer diagnosis and the ECT session was 7.3 ± 7.5 years (median 5 range 0–32). A total of 593 lesions were treated with ECT in this cohort with a mean size of 34 ± 65 mm (median 10 range 5-450). The mean number of metastases per patient was 3.0 ± 2.3 (median 3, range 1–7). The characteristics of the patient cohort and the treated lesions are reported in Table 1.

**Table 1:** Descriptive characteristics of patients (*N* = 195) and lesions (*N* = 593)

Patients’ variables		*N*	%	Lesions’ variables		*N*	%
Side	Right Left Both	82 106 7	42.1% 54.4% 3.6%	Electrode	Plate Row needles Hexagonal Finger	21 101 461 10	3% 17% 78% 2%
Tumour phenotype	Luminal a Luminal b Her2+ Triple neg Unknown	82 30 39 38 6	42.1% 15.4% 20.0% 19.5% 3.0%	Site	Chest wall Head/neck Abdomen Limbs Back	539 20 15 10 9	90.9% 3.4% 2.5% 1.7% 1.5%
Grading	1 2 3 Unknown	9 75 92 12	4.8% 39.9% 48.9% 6.4%	Pre-irradiated		240	39%
Histopathologic type	Ductal Lobular Other*	140 24 31	71.8% 12.3% 15.9%	Lymphoedema		50	8%
Distant metastases	Yes No	95 100	48.7% 51.3%	Ulcerated		92	15%
Skin nodule	Single nodule Multiple nodules	78 117	40% 60%	Size	≤ 3 cm > 3 cm	472 121	80% 20%
Concomitant systemic therapy	Chemotherapy Hormonotherapy Immunotherapy No	76 32 15 72	39.0% 16.4% 7.7% 36.9%				
Sites of metastases	Brain Lung Liver Bone Soft tissues Lymph nodes Contralateral	5 31 10 26 18 50 28	2.6% 16.1% 5.2% 13.5% 9.4% 25.9% 14.5%				

* Other: adenocarcinoma 6, angiosarcoma 4, infiltrating carcinoma 3, invasive apocrine carcinoma 2, micropapillary carcinoma 2, mucinosus carcinoma 1, Bowen disease 1, ductal-lobular 1, mesenchimal 1, neuroendocrine 1, unknown 9

The majority of patients had previously received standard oncologic treatments, including surgery (85.1%), systemic therapies (84.6%) and radiotherapy (46.7%). A high percentage of patients was also heavy pretreated: 32.3% had received more than 2 different cycles of systemic therapies, 8.2% had undergone more than 2 surgical interventions, and 2.6% had received more than 2 radiotherapy cycles. Patients were all treated with Bleomycin ECT.

Treatment response was evaluated at two months of follow-up, both per lesion and per patient. One-hundred and eight patients (55%) achieved CR, and 52 (27%) achieved PR (OR 82%). Twenty-four patients (12%) experienced SD, while 9 patients presented with PD (5%). Response evaluation was not possible in 2 patients due to skin crust formation. The response per lesion was: 65% CR, 21% PR (OR 86%), 11% SD, 2% PD, and 1% not evaluable.

### Toxicity and side effects

Pain intensity before ECT was mild (mean 1.8 ± 2.7) among patients and it significantly increased immediately after the procedure (mean 2.9 ± 3.1, *p* < 0.0001); it then decreased during the follow-up period to values comparable to baseline (mean 2.1 ± 2.8, 1.9 ± 2.6, 2.0 ± 2.6 at follow-up visits at 30, 90 and 150 days), which were not significantly different from pre-ECT levels. The percentage of patients with severe pain (VNS > 5) was 15% before ECT, which rose to 28% following the procedure (*p* = 0.0288), but decreased during follow-up (5% *p* = 0.0023, 6% *p* = 0.0125, 2% *p* = 0.0008). A significant decrease was noted in the percentage of patients with bleeding lesions, which dropped from 21% before ECT to 6% after 5 months (*p* = 0.0010). In contrast, a significant increase was observed in the percentage of patients with hyperpigmentation rising from 29% before ECT to 63% after 5 months (*p* < 0.0001). Severe symptomatology (grade 3–4) was reported in less than 10% of patients at follow-up, except for pruritus (13%), skin induration (13%), purpura (11%), skin atrophy (11%) and skin ulceration (11%).

### Analysis of objective response rate by receptor status

The analysis by receptor status, revealed a significantly different response rate among the subgroups (*p* = 0.0174). The objective response rate was 88% for Luminal A-like, 90% for Luminal B-like, 82% for HER2+, and 66% for Triple Negative subtype. The univariate logistic analysis showed a significant better response rate for Luminal A-like(*p* = 0.0060) and Luminal B like(*p* = 0.0271) groups compared to the Triple Negative group, and a significant worse response for Triple Negative group with respect to all the other groups (*p* = 0.0034).

### Analysis of objective response rate by number and size of lesions per patient

The patients were divided according to the number (single vs. multiple) and size of lesions (≤ 3 cm vs. > 3 cm). As a result, they were stratified into 4 groups: patients with a small (≤ 3 cm) and single lesion (*N* = 21), patients with a large (> 3 cm) and single lesion (*N* = 57), patients with small (≤ 3 cm) and multiple lesions (*N* = 80), patients with large (> 3 cm) and multiple lesions (*N* = 37).

The mean size of the “small single lesion” group was 18.8 ± 7.7 mm, while the mean size of the “large single lesion” group was 173.2 ± 111.6 mm (*p* < 0.0001). In the “small single lesion” group, 10% of lesions were ulcerated, compared to 54% of ulcerated lesions in “large single lesions” group (*p* = 0.0006). Objective response rates among the four subgroups were significantly different (*p* = 0.0017) as reported in Table 2. A further analysis of the multiple lesions groups showed that the objective response rate did not change with the increasing the number of lesions (*p* = 0.0973 for small multiple nodules, *p* = 0.0915 for large multiple nodules).

### Analysis of objective response rate by size and distant metastases

Similarly to the previous analysis, the cohort was divided into four groups based on tumor size (small nodules, large nodules) and presence of distant metastases (yes/no). The results were statistically significant (*p* = 0.0079) and are shown in Table 2.

**Table 2:** Response per patient in subgroups

Subgroups	*N*	ObjectiveResponse rate
Small single nodule (≤ 30 mm)	21	95%
Large single nodule (> 30 mm)	57	67%
Small multiple nodules (≤ 30 mm)	80	90%
Large multiple nodules (> 30 mm)	37	79%
Small nodules (≤ 30 mm) no mets	66	92%
Small nodules (≤ 30 mm) with mets	45	91%
Large nodules (> 30 mm) no mets	34	85%
Large nodules (> 30 mm) with mets	50	58%

### Uni- and multi- variate logistic analysis of objective response

The statistical relevance of a series of variables collected in the database was evaluated using univariate logistic analysis for objective response. After univariate analysis, only the significant variables were included in the multivariate model. The results are shown in Table 3.

**Table 3:** Uni- and multi- variate logistic analysis of the objective response

Variable	Univariate				Multivariate			
	OR	C.I.95%		*P* value	OR	C.I.95%		*P* value
		lower	upper			lower	upper	
Luminal score (0 = TN, 1 = HER2+, 2 = lumA, 3 = lumB) *	2.03	1.12	3.70	0.0201	1.45	0.67	3.15	0.3441
Grading	3.81	0.66	26.02	0.5427				
Age	1.03	1.01	1.05	0.0007	1.02	0.98	1.05	0.3225
Years since diagnosis	1.08	1.03	1.14	0.0026	1.04	0.95	1.14	0.3563
N surgery interventions	0.99	0.71	1.39	0.9826				
N systemic therapies cycles	0.97	0.83	1.14	0.7213				
N radiotherapy cycles	1.08	0.65	1.78	0.7767				
Time occurrence skin metastases	1.00	1.00	1.01	0.4165				
Distant metastases (no vs. yes)	3.21	1.45	7.13	0.0041	4.09	1.41	11.83	0.0095
Concurrent therapies (yes vs. no)	0.87	0.40	1.87	0.7213				
Systemic bleomycin injection vs. local	0.17	0.05	0.53	0.0027	0.82	0.17	3.85	0.8017
N lesions (multiple vs. single)	2.34	1.16	4.93	0.0246	1.70	0.58	4.92	0.3309
Lesion size (< 3 cm vs. > 3 cm)	5.08	2.23	11.58	0.0001	5.00	1.45	16.67	0.0106
Chest wall localisation (yes vs. no)	1.56	0.60	4.04	0.3604				
Preirradiated lesion (yes vs. no)	0.69	0.43	1.11	0.1243				
Lymphoedema (yes vs. no)	0.50	0.23	1.05	0.0665				
Deep margins treated (yes vs. no)	2.13	1.22	3.85	0.0080	1.32	0.23	7.63	0.7571
Lateral margins treated (yes vs. no)	2.08	1.19	3.57	0.0106	3.52	0.62	20.15	0.1573
Ulcerated lesion (yes vs. no)	0.51	0.28	0.91	0.0233	0.88	0.29	2.70	0.8138

* TN = triple negative, lumA = luminal A-like, lumB = luminal B-like

### Local progression free survival

**Fig. 1:**
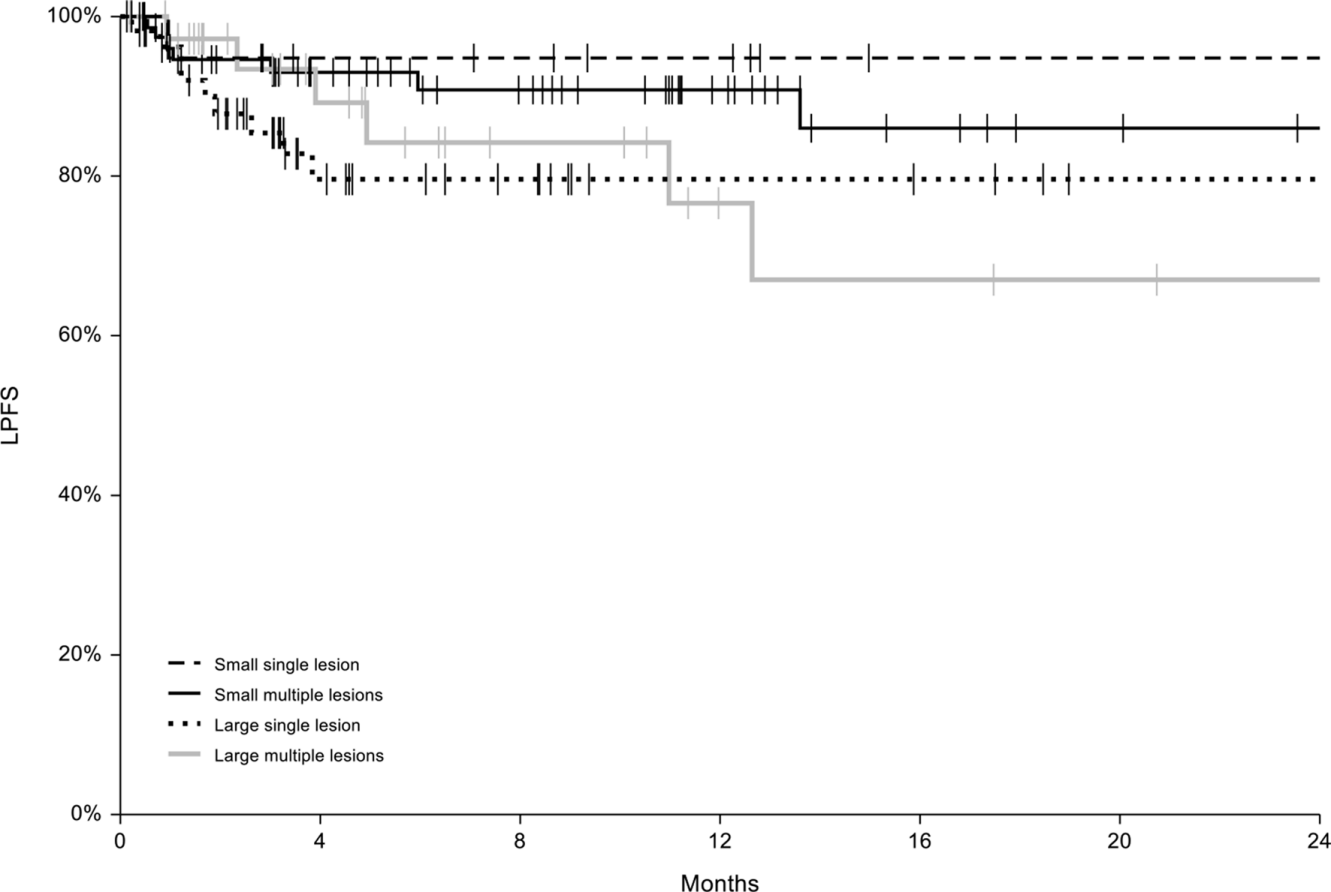
Local progression free survival (LPSF) curves for subgroups of patients

The mean follow-up time was 16 ± 17 months (median 11, range 5–78). During follow-up, 14 patients (7%) underwent a further ECT session to obtain a better response or to maintain their response over time. Cox regression analysis was applied to the entire cohort of patients to test the hazard ratios of a series of variables affecting local progression free survival. After univariate analysis, only the significant variables were included in the multi-variate model. The results are shown in Table 4. Local progression free survival (LPFS) curves were generated for four subgroups of patients based on the size and number of nodules. The curves are shown in Fig. 1 and are not significantly different.

One-year LPFS in the entire cohort was 86% (C.I. 82-89%). In the small single lesion group, it was 95% (C.I. 85-100%), in the large single lesion group it was 80% (C.I. 67-92%), in the small multiple lesions group 91% (C.I. 84-98%) and finally in the large multiple lesions group 77% (C.I. 57-96%). The two years LPFS for the entire cohort was 79% (C.I. 70-88%).

**Table 4:** Cox regression analysis of local progression free survival in the cohort of patients

Variable	Univariate				Multivariate			
	RR	C.I.95%		*P* value	RR	C.I.95%		*P* value
		lower	upper			lower	upper	
Luminal score (0 = TN, 1 = HER2+, 2 = lum A, 3 = lum B) *	2.34	1.17	4.70	0.0162	1.76	0.83	3.74	0.1430
Grading	2.12	0.92	4.87	0.0765				
Age	0.96	0.93	0.99	0.0055	0.96	0.92	0.99	0.0202
Years since diagnosis	0.96	0.90	1.03	0.2394				
N surgery interventions	1.04	0.76	1.42	0.8199				
N systemic therapies cycles	0.92	0.73	1.13	0.4035				
N radiotherapy cycles	1.21	0.73	1.99	0.4623				
Time occurrence skin metastases	1.00	1.00	1.00	0.2507				
Distant metastases (yes vs. no)	0.37	0.15	0.90	0.0274	0.96	0.36	2.57	0.9289
Concom therapies (yes vs. no)	1.23	0.51	2.96	0.6496				
Systemic bleomycin injection vs. local	0.97	0.33	2.84	0.9569				
N lesions (multiple vs. single)	0.89	0.39	1.99	0.7685				
Lesions’ size (< 3 cm vs. > 3 cm)	2.65	1.15	6.10	0.0217	2.47	0.85	7.19	0.0977
Chest wall localisation (yes vs. no)	1.84	0.25	13.62	0.5512				
Preirradiated lesion (yes vs. no)	0.51	0.23	1.15	0.1055				
Lymphoedema (yes vs. no)	0.84	0.29	2.44	0.7424				
Deep margins (yes vs. no)	2.29	0.91	5.78	0.0784				
Lateral margins (yes vs. no)	2.95	1.10	7.91	0.0314	2.05	0.73	5.77	0.1722
Ulcerated lesion (yes vs. no)	0.45	0.19	1.04	0.0608				
Response to ECT (OR vs. no)	6.13	3.88	9.71	0.0000	7.79	4.32	14.03	0.0000

* TN = triple negative, lumA = luminal A-like, lumB = luminal B-like

RR = relative risk, OR = objective response,

### Overall survival analysis

**Fig. 2:**
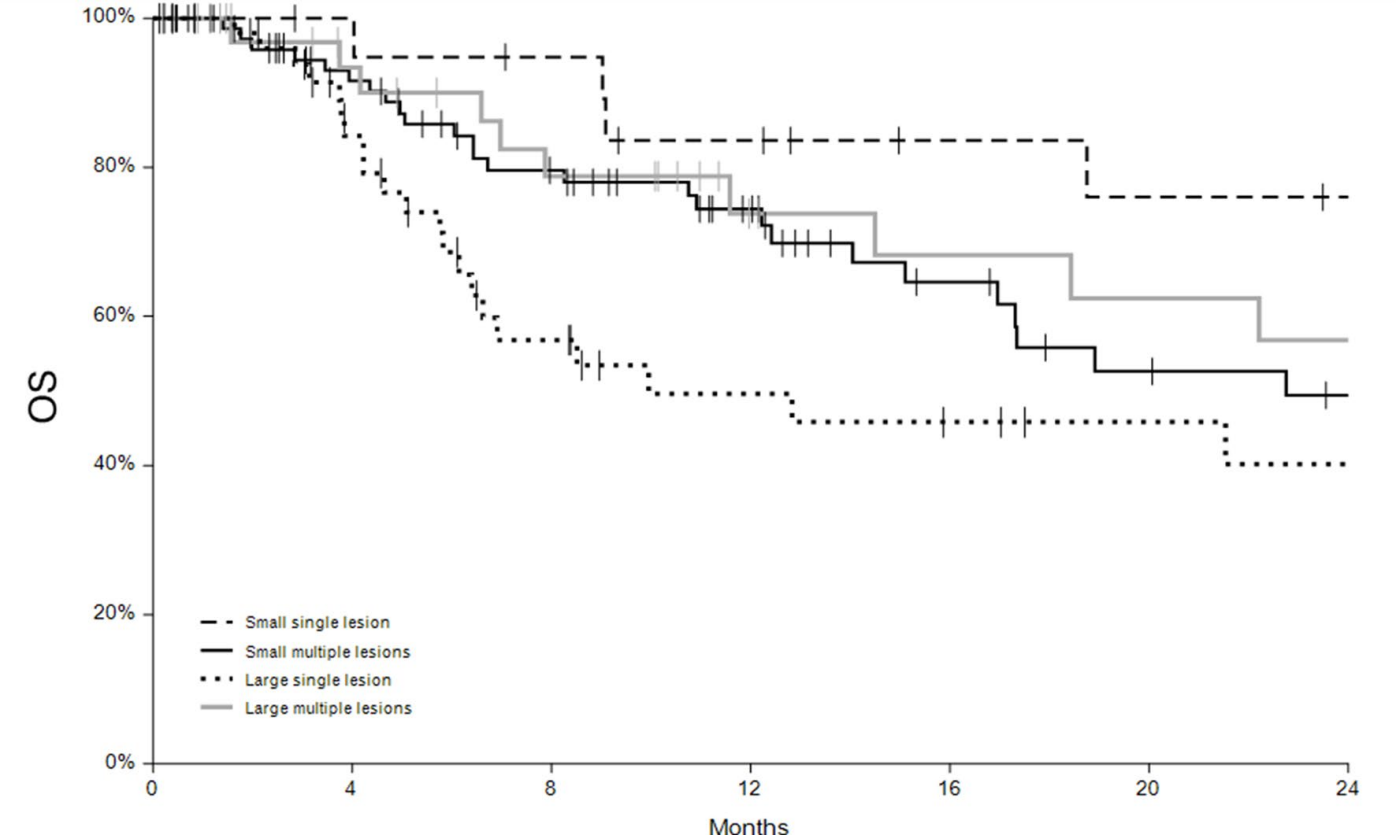
Overall survival curves for subgroups of patients

During the follow-up period, 77 patients died (39%) after a mean time of 14 ± 14 months (median 8.3 months, range 1.4–69.6). The one-year overall survival rate was 69% (C.I. 62-77%), and the 2-year overall survival rate was 52% (C.I. 43-62%). Survival curves of the four subgroups of patients based on tumor size and number are shown in Fig. 2. Significant differences were observed among groups, particularly between the large single nodule and large multiple nodules groups (*p* = 0.0436) and between small and large single nodule groups (*p* = 0.0157). The overall survival rates were 84% (C.I. 67-100%) for patients with small single nodules, 74% (C.I.64-85%) for patients with small multiple nodules, 74% (C.I. 57-91%) in patients with large multiple nodules and 50% (C.I. 33-66%) in patients with large single nodules. Cox regression analysis was applied to the entire cohort of patients to assess the risk ratios of various variables affecting overall survival. After uni-variate analysis, the significant variables identified were time between diagnosis and ECT (*p* = 0.0240), absence of distant metastases (*p* = 0.0181), absence of ulceration (*p* = 0.0379) and positive response to ECT (*p* < 0.0001).

## Discussion

This multicentre prospective study confirms the efficacy and safety of ECT in the treatment of skin metastases from breast cancer and provide further insight on how to select candidate patients. Of note, response to ECT is associated with improved superficial disease control and survival, suggesting the importance of early introduction of this form of locoregional treatment in the management of breast cancer patient with cutaneous metastases. Although this study did not meet the target accrual to confirm the results of the retrospective GISEL study [17], its results point to the same direction and suggest a higher sensitivity of luminal A-like cancers to ECT.

The inclusion criteria of the study foresaw the enrolment of patients with advanced breast cancer, presenting with local and/or distant, single or multiple cutaneous or subcutaneous metastases, even in presence of other distant metastatic sites. As a result, all enrolled patients had undergone extensive pre-treatment, including multiple surgeries, radiotherapy, and several lines of systemic treatments such as chemotherapy, hormone therapy, immunotherapy and targeted therapy. Patients were treated with ECT for cutaneous or subcutaneous metastases when no other local standard treatments were feasible, tolerated or indicated.

The analysis of the objective response rate revealed that Luminal A-like and Luminal B-like patients exhibited better objective response rates (88% and 90%, respectively) compared to HER2 + and Triple Negative patients (82% and 66%, respectively). Although a larger number of patients would be required to confirm this hypothesis, to our knowledge, this study is based on the largest cohort study of breast cancer patients treated with ECT so far. The response observed for Luminal A-like and Luminal B-like patients are similar to those reported in the previous study [17] and in the study by Wichtowski et al. [23], while the results obtained for HER2 + and Triple Negative groups show a lower objective response rate. A similar trend, with lower objective response rate in Triple Negative subset of patients, was observed by Di Prata et al. [24]. In their cohort of 177 patients, those with Triple Negative subtype showed an objective response rate of 64%, compared to 86% in HER2 + patients and 80% in HR + patients. Although the response to ECT appears to be lower in Triple-Negative patients, it is still sustained, as shown by a positive outcome in 66% of patients. This result is clinically relevant, considering that systemic therapy alone may offer limited benefit to the patients with skin metastases, both in terms of clinical response and symptom palliation [25].

One of the most frequently raised question by the oncology community relates to ECT selection criteria. We attempted to address this by conducting a series of sub-analysis on our patient cohort.

We applied univariate and multivariate logistic models to identify the significant variables predicting a response. In the univariate model, several factors showed a significant association with treatment outcome, including receptor status, patient age, time since breast cancer diagnosis, presence of distant metastases, route of drug administration, number of skin metastases, tumor size, coverage of deep and lateral margins, and tumor ulceration. Given that many of these factors are interrelated a multivariate analysis was also performed. The multivariate model revealed that the factors independently associated with response to ECT were lesion size and the presence of distant metastases. As ECT is a highly effective local therapy for small and multiple lesions on the skin and in the subcutaneous area [17, 23, 24, 26–30], we proceed to analyse four subgroups of patients: those with a single small lesion; those with a single large lesion; those with multiple small lesions; finally, those with multiple and large lesions.

As expected, we observed significantly different outcomes, with higher response rates in patients with small lesions, either single or multiple (OR 95% and 90%, respectively). Even the patients with large multiple metastases achieved a favourable outcome with an OR of 79%. The poorest results were noted in those with large single lesions (mean size 17 cm). Although the aim of treatment in the latter cases is symptom relief, the OR rate was 67%. Particularly in the case of uncomplete response, it is important to evaluate treatment outcome in terms of symptom relief. In these regard, even a partial response might be clinically relevant as long as it translates into a benefit for the patient’s quality of life by alleviating ulceration, halting bleeding and decreasing pain [16, 31, 32].

As to patient selection criteria, small tumor size appears to be consistently associated with better response to ECT, regardless the presence of distant metastases as shown by the excellent outcome of patients with either single or multiple metastases (OR 92% and 91%, respectively).

Patients presenting with large lesions and no other metastatic sites showed an OR of 85%. However, those with large cutaneous lesions and distant metastases showed a significantly lower response to ECT, with an OR of 58%. Conversely, ECT proved to be highly effective in small lesions, even in the presence of distant metastases. This observation might suggest independent pathways of disease progression in the skin compared to other organs and aligns with findings that various systemic treatments for breast cancer are effective in preventing visceral disease progression but fail to achieve substantial control over skin and subcutaneous tissue involvement. This disparity underscores the impact of intra-tumor heterogeneity, which is a significant factor contributing to therapeutic resistance and is a primary reason for the poor clinical outcomes seen in metastatic cancer patients [33]. Tumor heterogeneity, stemming from the genomic and molecular peculiarities of different tumour cell populations, explains the different clinical manifestations of the disease between the various metastatic sites with its impact becoming more pronounced as the disease burden increase [34]. Therefore, when systemic treatment is effective, the concurrent use of ECT can promote local control of skin metastasis [35]. Grischke et al. demonstrated that combining local and systemic therapies results in acceptable toxicity, concluding that ECT serves as an effective adjunct to systemic therapy, especially for patients with progressive cutaneous disease, without causing major systemic or loco-regional toxicities [35]. Most importantly, achieving local control of skin lesions can enhance the quality of life of these patients, who often experience issues such as unpleasant odour, pain, bleeding, ulceration, and psychological distress [24].

One of the aims of this study was to assess the impact of ECT on LPFS and OS. The one-year LPFS in the cohort of patients was 86% (C.I. 82-89%) and two-year LPFS was 79% (C.I. 70-88%), with small non-significant differences among subgroups. In the small and single lesion group, the one-year LPFS was 95% (C.I. 85-100%), in the large and single lesion group it was 80% (C.I. 67-92%), in the small and multiple lesions group it was 91% (C.I. 84-98%) and finally in the large and multiple lesions group it was 77% (C.I. 57-96%). The univariate Cox regression analysis identified several significant predictors of longer LPFS including receptor status, younger age, absence of distant metastases, small lesions size, lateral margins coverage during treatment and objective response to ECT. In the multivariate analysis, age and objective response to ECT were confirmed as significantly associated with longer LPFS. These results corroborate the findings from our previous retrospective study, where one-year LPFS was 86.2% [10]. Moreover, small nodule size, lack of ulceration, absence of distant metastases and receptor status (in particular, luminal A-like and luminal B-like), were significantly associated with better LPFS. Interestingly, the presence of concomitant systemic treatment does not seem to affect the local disease control at the cutaneous level. However, the patient population included in the study is heavily pretreated with 32.3% having received more than 2 lines of therapy, thus the expected clinical benefit from a second- or third-line systemic therapy is still very low.

In the study published by the INSPECT group, one-year LPFS was higher for HER2 + and HR + patients compared to Triple Negative patients, which aligns to our findings. Furthermore, a sub-analysis conducted in these subgroups revealed that, in HR + group, small lesions size is an indicator of a longer progression free survival; in contrast, in Triple Negative patients, a lower number of lesions and presence of concomitant treatments positively influence the progression free survival. Additionally, one-year progression free survivals rates ranged from 61% in Triple Negative patient to 81% in HR + group, which are slightly lower than those observed in our study [24]. This is likely due to the longer follow-up time of our study and to the progressively earlier indication for ECT in patients with cutaneous or subcutaneous metastases which naturally occurs as clinicians become more aware of the beneficial impact of therapy on patients. In fact, the study by Di Prata reported a higher percentage of large lesions (> 3 cm) compared to our work. The LPFS values observed here are comparable to those reported by Campana et al. [36], who analysed young (< 70 years) and elderly (≥ 70 years) metastatic breast cancer patients with two-year LPFS ranging from 67 to 93%. In our study, the mean age of the cohort was 67 years with a median age of 69, indicating an almost symmetrical age distribution around 69 years of age, with a two-year LPFS of 79%.

Our study demonstrates that a positive outcome (complete or partial response) to ECT has an impact on overall survival (OS). This correlation suggests that a better response to locoregional treatment, especially those with oligometastatic disease, may provide a survival benefit.

Of note, recent studies would suggest the value of ECT in combination with systemic treatment to improve patient OS [24, 37]. However, this result should be carefully interpreted, given the heterogeneity of our study population in terms of receptor profile, previous treatments, concomitant systemic therapies, and subsequent therapies following ECT; additionally, a significant portion of patients (51%) had cutaneous disease without other metastatic sites. Moreover, the interactions between the immune system, concomitant therapies or previous treatments (immunotherapy, chemotherapy or targeted therapy) and ECT are not yet well established and further studies are needed.

This prospective study has some limitations, the most notable is the relatively small number of patients recruited compared to the intended target, largely due to the constraints imposed by the COVID-19 pandemic. Second, the duration of the follow-up was relatively short (median 11 months). Finally, the inclusion of quality of life results would provide a more detail picture of patient outcome.

Nonetheless, this study confirms that ECT is a safe and effective treatment option for patients with advanced breast cancer and cutaneous metastases, with minimal toxicity. ECT is low demanding for the patients and can be safely applied not only as a palliative measure but also as an alternative treatment for patients who are not eligible for standard treatments, or in combination with them [17, 24, 29, 35, 37].

The patients who benefit most from the treatment are those with small lesions, either single or multiple, regardless of the presence of distant metastases. Moreover, even patients with large lesions may achieve sustained response and disease control. In this regard, it is also important to recognize the role of ECT in treating large single lesions, known as “armor lesions”, in which it can alleviate symptoms such as pain, bleeding, ulceration, odour and infections. Skin involvement is a relatively common occurrence in the metastatic progression of breast cancer, affecting up to 30% of advanced cases in various studies [7, 38]. Since skin metastases are constantly visible to the patient, they often cause strong psychological distress [31]. Reducing the size and symptoms in patients with large metastatic lesions can greatly improve their quality of life and psychological well-being.

## Data Availability

Clinical records were anonymously entered into a dedicated encrypted online database. The database is available, upon request, at IOV specific repository.
